# Slip Effects on Peristaltic Transport of a Particle-Fluid Suspension in a Planar Channel

**DOI:** 10.1155/2015/703574

**Published:** 2015-06-02

**Authors:** Mohammed H. Kamel, Islam M. Eldesoky, Bilal M. Maher, Ramzy M. Abumandour

**Affiliations:** ^1^Department of Engineering Mathematics and Physics, Faculty of Engineering, Cairo University, Giza, Egypt; ^2^Department of Basic Engineering Sciences, Faculty of Engineering, Menoufia University, Shebin El-Kom, Egypt

## Abstract

Peristaltic pumping induced by a sinusoidal traveling wave in the walls of a two-dimensional channel filled with a viscous incompressible fluid mixed with rigid spherical particles is investigated theoretically taking the slip effect on the wall into account. A perturbation solution is obtained which satisfies the momentum equations for the case in which amplitude ratio (wave amplitude/channel half width) is small. The analysis has been carried out by duly accounting for the nonlinear convective acceleration terms and the slip condition for the fluid part on the wavy wall. The governing equations are developed up to the second order of the amplitude ratio. The zeroth-order terms yield the Poiseuille flow and the first-order terms give the Orr-Sommerfeld equation. The results show that the slip conditions have significant effect within certain range of concentration. The phenomenon of reflux (the mean flow reversal) is discussed under slip conditions. It is found that the critical reflux pressure is lower for the particle-fluid suspension than for the particle-free fluid and is affected by slip condition. A motivation of the present analysis has been the hope that such theory of two-phase flow process under slip condition is very useful in understanding the role of peristaltic muscular contraction in transporting biofluid behaving like a particle-fluid mixture. Also the theory is important to the engineering applications of pumping solid-fluid mixture by peristalsis.

## 1. Introduction

Peristalsis is a form of a fluid transport induced by a progressive wave of area contraction or expansion along the walls of a distensible duct containing liquid. In physiology, peristaltic mechanism is involved in many biological organs such as ureter, gastrointestinal tract, ducts afferents of the male reproductive tracts, cervical canal, female fallopian tube, lymphatic vessels, and small blood vessels. In addition, peristaltic pumping occurring in many practical applications involving biomechanical systems such as roller, finger pumps, and heart-lung machine have been fabricated.

Since the first investigation of Latham [1], several theoretical and experimental studies have been conducted in the past to understand peristaltic action in different situations.

The literature on peristalsis is by now quite extensive, and a summary of most of the investigations has been presented in detail by Rath [2], L. M. Srivastava and V. P. Srivastava [3–5], Srivastava and Saxena [6], and V. P. Srivastava and L. M. Srivastava [7].

The theoretical study of the theory of particle-fluid mixture is very useful in understanding a number of diverse physical problems concerned with powder technology, fluidization, transportation of solid particles by a liquid, transportation liquid slurries in chemical and nuclear processing, and metalized liquid fuel slurries for rocketry. The sedimentation of particles in a liquid is of interest in much chemical engineering process, in medicine, where erythrocyte sedimentation has become a standard clinical test, and in oceanography as well as other fields. The particulate theory of blood has recently become the object of scientific research, Hill and Bedford [8], L. M. Srivastava and V. P. Srivastava [3–5, 9], Trowbridge [10], and Oka [11]. A number of research works on the topic, with and without peristalsis, have been reviewed by L. M. Srivastava and V. P. Srivastava [5]. Applications of the theory of particle-fluid mixture to the microcirculation and erythrocyte sedimentation included the work of Wang and Skalak [12], Bungay and Brenner [13], Skalak et al. [14], and Karino et al. [15].

Peristaltic transport of solid particle with fluid was first attempted by Hung and Brown [16]. They initiated the study of the peristaltic transport of solid particles, which included an experimental work on the particle transport in two-dimensional vertical channels having various geometries. In this connection also another paper by Brown and Hung [17] and a study by Takabatake and Ayukawa [18] are worth mentioning. Both studies have employed finite difference technique to solve two-dimensional nonlinear peristaltic flows problem. L. M. Srivastava and V. P. Srivastava [5] studied the peristaltic pumping of a particle-fluid mixture in a two-dimensional channel carried out mathematically; a perturbation solution is obtained. Mekheimer et al. [19] studied the peristaltic pumping of a particle-fluid suspension in a planar channel. El Misery et al. [20] studied the peristaltic motion of an incompressible generalized Newtonian fluid in a planar channel.

No-slip boundary conditions are convenient idealization of the behavior of viscous fluids near walls. The inadequacy of the no-slip condition is quite evident in polymer melts which often exhibit microscopic wall slip. The slip condition plays an important role in shear skin, spurt, and hysteresis effects. The boundary conditions relevant to flowing fluids are very important in predicting fluid flows in many applications. The fluids that exhibit boundary slip have important technological applications such as in polishing valves of artificial heart and internal cavities [21]. The slip effects on the peristaltic flow of a non-Newtonian Maxwellian fluid have been investigated by Eldesoky [22]. The influence of slip condition on peristaltic transport of a compressible Maxwell fluid through porous medium in a tube has been studied by Chu and Fang [23]. Many recent researches have been made in the subject of slip boundary conditions [24–33].

From the previous studies, there is no any attempt to study the effect of slip condition on the flow of a particle-fluid suspension with peristalsis. The purpose of this paper is to study the slip effects on the peristaltic pumping of a particle-fluid mixture in a two-dimensional channel. It is an application of the two-dimensional analysis of peristaltic motion of a particle-fluid mixture by L. M. Srivastava and V. P. Srivastava [5] and the two-dimensional analysis of peristaltic motion of single phase fluid by Fung and Yih [34] in the presence of slip effect. The mathematical model considers a particle-fluid mixture between infinite parallel walls with slip condition on which a sinusoidal traveling wave is imposed. A perturbation solution is obtained which satisfies the momentum equations for the case in which amplitude ratio (wave amplitude/channel half width) is small. Finally, the phenomenon of the mean flow reversal is presented and its physiological implication is discussed. Beside the engineering applications of pumping particle-fluid mixture by peristalsis, the present analysis of two-phase flow process is potentially important in regard to biofluid transport by peristalsis muscular contractions in body organs where fluids behave like particle-fluid mixtures, namely, chime in small intestine, spermatic fluid in cervical canal, urine (from a diseased kidney) in ureter, and blood suspension in arteriole.

## 2. Formulation of the Problem

**Figure 1:**
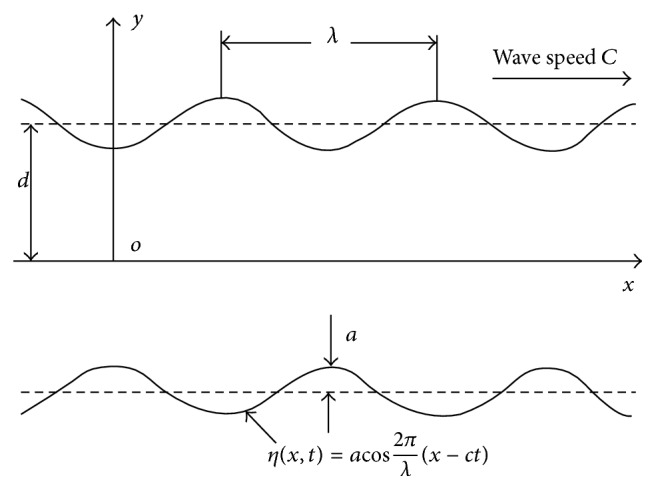
Geometry of the problem.

Consider a two-dimensional infinite channel of mean width 2*d* (see Figure 1), filled with a mixture of small spherical rigid particles in an incompressible Newtonian viscous fluid. The walls of the channel are flexible, on which are imposed travelling, sinusoidal wave of small amplitude. The equations governing conservation of mass and linear momentum for both fluid and particle phase using a continuum approach are expressed as follows (Drew [35]; L. M. Srivastava and V. P. Srivastava [5, 9]).

*Fluid Phase*

(1) 1−Cρf∂uf∂t+uf∂uf∂x+υf∂uf∂y=−1−C∂p∂x+1−CμsC∇2uf+CSup−uf,

(2) 1−Cρf∂vf∂t+uf∂vf∂x+vf∂vf∂y=−1−C∂p∂y+1−CμsC∇2vf+CSvp−vf,

(3) ∂∂x1−Cuf+∂∂y1−Cvf=0.

*Particulate Phase*

(4) Cρp∂up∂t+up∂up∂x+υp∂up∂y=−C∂p∂x+CSuf−up,

(5) Cρp∂vp∂t+up∂vp∂x+υp∂vp∂y=−C∂p∂y+CSvf−vp,

(6) ∂∂xCup+∂∂yCvp=0.

In (1)–(6), *x* and *y* are Cartesian coordinates with *x* measured in the direction of wave propagation and *y* measured in the direction normal to the mean position of the channel walls, (*u*_*f*_, *v*_*f*_) denotes fluid phase velocities, (*u*_*p*_, *v*_*p*_) denotes particulate phase velocities, *ρ*_*f*_ and *ρ*_*p*_ are the actual densities of the materials constituting fluid and particulate phase, respectively, (1 − *C*)*ρ*_*f*_ is the fluid phase density, *Cρ*_*p*_ is the particulate phase density, *p* denotes the pressure, *C* denotes the volume fraction density of the particles, *μ*(*C*) is the particle-fluid mixture viscosity (also the* effective viscosity* of the suspension), and *S* the drag coefficient of interaction for the force exerted by one phase on the other.

The concentration of the particles is considered to be so small that the field interaction between particles may be neglected. Thus, the diffusivity terms, which can model the effects of particle-particle impacts due to the Brownian motion, are neglected. It is worth mentioning here that the effect of Brownian motion was considered by others including Batchelor [36, 37]. The volume fraction density, *C*, of the particles is chosen also constant. This is a good assumption for low concentration of small particles.

The expression for the drag coefficient for the present problem is selected as

(7) S=92μ0a2λ′C,λ′C=4+38C−3C21/2+3C2−3C2,

where *μ*_0_ is the fluid viscosity and *a* is the radius of the particle. Relation (7) represents the classical Stokes' drag for small particle Reynolds number, modified to account for the finite particulate fractional volume through the function *λ*′(*C*), obtained by Tam [38].

Many empirical relations have been suggested to express the viscosity of the suspension as a function of particle concentration and viscosity of the suspending medium. Einstein was the first to obtain theoretically that the viscosity of the suspension *μ*_*S*_ was related to that of the suspending medium *μ*_0_ for spheres in suspension by *μ*_0_ = *μ*_*S*_(1 − 2.5*C*). However, the Einstein formula expresses the viscosity of the suspension only when *C* is less than 0.05. As *C* increases from 0.05, the suspension viscosity varies from the Einstein equation. For the present problem, an empirical relation for the viscosity of the suspension is as follows:

(8a) μSC=μ011−qC,

(8b) q=0.07exp2.49C+1107Texp−1.69C,

where *T* the absolute temperature (°K), suggested by Charm and Kurland (1974) [39], is used. The viscosity of the suspension expressed by this formula is found to be reasonably accurate up to *C* = 0.6. Charm and Kurland (1974) [39] tested (8a) and (8b) with a cone and plate viscometer and found it to be in agreement within* ten *percent in case of blood suspension.

The boundary conditions that must be satisfied by the fluid on the walls are the slip and impermeability conditions. The walls of the channel are assumed to be flexible but extensible with a travelling sinusoidal wave, and displacement in the channel walls is in transverse direction only. Hence, boundary conditions are

(9a) ufΨfy=∓A∂uf∂y,

(9b) vf−Ψfx=±∂η∂ton⁡  y=±d±η,

(9c) vp−Ψpx=±∂η∂t,

where Ψ, the stream function, is such that

(10) uf=∂Ψf∂y,vf=−∂Ψf∂x,up=∂Ψp∂y,vp=−∂Ψp∂x.

The transverse displacement, *η*, of the wall is represented as

(11) $$\begin{array}{c} \eta \left(\;x,t\;\right)=a\cos\frac{2\pi }{\lambda }\left(\;x-ct\;\right), \end{array}$$

where *a* is the amplitude, *λ* the wavelength, and *c* the wave speed.

We now select the following set of nondimensional variables and parameters:

(12) x′=xd,y′=yd,uf′=ufc,vf′=vfc,up′=upc,vp′=vpc,η′=ηd,Ψf′=Ψfcd,Ψp′=Ψpcd,t′=ctd,p′=pρfc2,υ=μ0ρf.

Suspension Reynolds number

(13) $$\begin{array}{c} R=\frac{cd\rho_{f}}{\left(1-C\right)\mu_{S}}. \end{array}$$

Wave number

(14) $$\begin{array}{c} \alpha =\frac{2\pi d}{\lambda }. \end{array}$$

Knudsen number

(15) $$\begin{array}{c} Kn=\frac{A}{R}. \end{array}$$

Amplitude ratio

(16) $$\begin{array}{c} \varepsilon =\frac{a}{d}. \end{array}$$

Suspension parameter

(17) $$\begin{array}{c} M=\frac{Sd^{2}}{\left(1-C\right)\mu_{S}}. \end{array}$$

Suspension parameter

(18) $$\begin{array}{c} N=\frac{Sd^{2}\rho_{f}}{\left(1-C\right)\rho_{p}\mu_{S}}. \end{array}$$

Thus, the systems of (1)–(6), and (9a)–(11) now become, after dropping the primes,

(19) 1−CR∂∂t∇2Ψf+Ψfy∇2Ψfx−Ψfx∇2Ψfy=∇4Ψf+CM∇2Ψp−∇2Ψf,

(20) CR∂∂t∇2Ψp+Ψpy∇2Ψpx−Ψpx∇2Ψpy=CN∇2Ψf−∇2Ψp,

(21) η=εcos⁡αx−t,

(22) Ψfy=∓KnΨfyy,

where ∇^2^ denotes the Laplacian operator.

## 3. Method of Solution

Assuming the amplitude ratio *e* of the wave is small, we obtain the solution for the stream function as a power series in terms of *ε*, by expanding Ψ_*f*_, Ψ_*p*_, and ∂*p*/∂*x* in the form (Fung and Yih (1968) [34])

(23) Ψf=Ψf0+εΨf1+ε2Ψf2+⋯,

(24) Ψp=Ψp0+εΨp1+ε2Ψp2+⋯,

(25) ∂p∂x=∂p∂x0+ε∂p∂x1+ε2∂p∂x2+⋯

In (25), the first term on the right-hand side corresponds to the imposed pressure gradient associated with the primary flow and the other terms correspond to the peristaltic motion or higher imposed pressure gradient.

Substituting (23) and (24) in (19), (20), and (22) and collecting terms of like powers of  *ε*, we obtain three sets of coupled linear differential equations with their corresponding boundary conditions in Ψ_(*f*,*p*)0_, Ψ_(*f*,*p*)1_, and Ψ_(*f*,*p*)2_, for the first three powers of *ε*.

The first set of differential equations in Ψ_(*f*,*p*)0_, subject to the steady parallel flow and transverse symmetry assumption for a constant pressure gradient in the *x*-direction, yields the following classical Poiseuille flow for the fluid and the particulate phase:

(26) Ψf0=Ky−13y3+2Kn2+Kny−12y2,

(27) Ψp0=Ky+2yM−13y3,

(28) K=−R2∂p∂x0,

where *K* is the Poiseuille flow parameter.

Thus, the effect of the particles on the fluid velocity profile is to cause an increase in the viscosity; that is, fluid viscosity *μ*_0_ is replaced by suspension viscosity (see (8a) and (8b)), and thus for a given pressure difference less fluid will flow through the channel. Further, the particles lead the fluid by a relative velocity proportional to 1/*M*(∂*p*/∂*x*)_0_.

The second and third sets of differential equations in Ψ_(*f*,*p*)1_ and Ψ_(*f*,*p*)2_ with their corresponding boundary conditions are satisfied by

(29a) 2Ψf1x,y,t=Φf1yeiαx−t+Φf1∗ye−iαx−t,

(29b) 2Ψp1x,y,t=Φp1yeiαx−t+Φp1∗ye−iαx−t,

(30a) 2Ψf2x,y,t=Φf20+Φf22ye2iαx−t+Φf22∗ye−2iαx−t,

(30b) 2Ψp2x,y,t=Φp20+Φp22ye2iαx−t+Φp22∗ye−2iαx−t.

A substitution of (29a), (29b) and (30a), (30b) into the differential equations and their corresponding boundary conditions in Ψ_(*f*,*p*)1_ and Ψ_(*f*,*p*)2_ leads to the following set of differential equations:

(31) d2dy2−α2+i1−C·αR1−K1−y2+2Kn2+Kn1−y·d2dy2−α2Φf1−2iK1−CαRΦf1=CMd2dy2−α2Φf1−Φp1,

(32) iCαR1−K1−y2+2M+2Kn2+Kn1−y+2M·d2dy2−α2Φp1−2iKCαRΦp1=CNd2dy2−α2Φp1−Φf1,

(33a) Φf1′±1−2K∓2KKn2+Kn=∓KnΦf1′′±1∓2K,

(33b) Φf1±1=±1,

(33c) Φp1±1=±1,

(34) Φf20′′′′y=−1−CαR2Φf1Φf1∗′′−Φf1∗Φf1′′′+CMΦf20′′−Φp20′′,

(35) 0=−iCαR2Φp1Φp1∗′′−Φp1∗Φp1′′′+CNΦp20′′−Φf20′′,

(36) d2dy2−4α2d2dy2−4α2+2i1−CαRΦf22=2i1−CKαR1−y2+2Kn2+Kn1−y·d2dy2−4α2Φf22+4i1−CKαRΦf22+i1−CαR2Φf1′Φf1′′−Φf1Φf1′′′+CMd2dy2−4α2Φf22−Φp22,

(37) 2iCαRd2dy2−4α2Φp22=2iCKαR1−y2−2M×d2dy2−4α2Φp22+4iCKαRΦp22+iCαR2Φp1′Φp1′′−Φp1Φp1′′′+CNd2dy2−4α2Φp22−Φf22,

(38a) Φf20′±1±12Φf1′′±1+Φf1∗′′±1−K=∓KnΦf20′′±1±12Φf1′′′±1+Φf1∗′′′±1,

(38b) Φf22′±1±12Φf1′′±1−K2=∓KnΦf22′′±1±12·Φf1′′′±1,

(38c) Φf22±1±14Φf1′±1=0,

(38d) Φp22±1±14Φp1′±1=0.

Thus, we obtained a set of differential equations together with the corresponding boundary conditions which are sufficient to determine the solution of the problem up to the second order in *ε*. Now, our main attention is to find out solution of differential equations for *Ф*_*f*1_ and *Ф*_*p*1_. Although (31) and (32) for *Ф*_*f*1_ and *Ф*_*p*1_ are coupled fourth-order ordinary differential equations with variable coefficients, it would, perhaps, be impossible to obtain solution of these differential equations for arbitrary values of *R*, *α*, and *K*. This is just because of the moving boundary considered in the present problem. The condition of moving boundary has made the boundary condition nonhomogeneous and thus the problem is not an eigenvalue problem as in all problems of hydrodynamic stability for which solutions are available in the literature. However, we can restrict our investigation to the case of pumping of an initially stagnant fluid, corresponding to no imposed pressure gradient. Thus, in this case (∂*p*/∂*x*)_0_ = 0, which means that constant *K* vanishes and we would be able to obtain a simple closed form analytical solution of this interesting case of free pumping. Physically, this assumption means that the fluid is stationary if there are no peristaltic waves. In fact, this assumption is not so restrictive because the maximum pressure gradient that small-amplitude waves can generate is of the order of *ε* ^2^ and in the pumping range the zeroth-order mean pressure gradient must certainly vanish.

Solutions of (31) and (32) subject to the boundary condition ((33a), (33b), and (33c)), under the assumption, *K* = 0, may be obtained as

(39) Φf1y=A1sinh⁡αy+B1sinh⁡βy,Φp1y=A2sinh⁡αy+B2sinh⁡βy,

where

(40) β2=α2−iαR1−C+CMN−iαR.A1=−βcosh⁡β+Knβ2sinh⁡βαcosh⁡αsinh⁡β−βcosh⁡βsinh⁡α+Kn sinhαsinh⁡βα2−β2,B1=αcosh⁡α+Knα2sinh⁡ααcosh⁡αsinh⁡β−βcosh⁡βsinh⁡α+Kn sinhαsinh⁡βα2−β2,A2=1−B2sinh⁡βsinh⁡α,B2=B1NN−iαR.

Next, in the expansion of Ψ_(*f*,*p*)2_, we need only to concern ourselves with the terms Φ_(*f*,*p*)20_(*y*) as our aim is to determine the mean flow only. Thus, the solution of the coupled differential equations ((34), (35)) subject to the boundary conditions ((38a), (38b), (38c), and (38d)), under the assumption, *K* = 0, gives the expressions

(41) Φf20′y=Fy−F1+D−C11−y2+2Kn,Φp20′y=Gy−F1+D−C11−y2+2M+2Kn,

where

(42) D=Φf20′±1=−12A1+A1∗α2sinh⁡α+α3Kn  coshα+B1β2sinh⁡β+β3Kn coshβ+B1∗β∗2sinhβ∗+β∗3cosh⁡β∗,Fy=1−C2α2R24γ2A1∗B1+CMA2∗B21−CN×cosh⁡α+βyα+β2−cosh⁡α−βyα−β2+γ∗2A1B1∗+CMA2B2∗1−CN×cosh⁡α+β∗yα+β∗2−cosh⁡α−β∗yα−β∗2+γ2+γ∗2×B1B1∗+CMB2B2∗1−CNcosh⁡β+β∗yβ+β∗2−cosh⁡β−β∗yβ−β∗2,Gy=Fy−1−C2α2R24Nγ2A2∗B2cosh⁡α+βy−cosh⁡α−βy+γ∗2A2B2∗cosh⁡α+β∗y−cosh⁡α−β∗y+γ2+γ∗2·B2B2∗cosh⁡β+β∗−cosh⁡β−β∗,

with

(43) $$\begin{array}{c} \gamma^{2}=1+\frac{CM}{\left(1-C\right)\left(N-i\alpha R\right)}. \end{array}$$

Thus, we see that one constant *C*_1_ remains arbitrary in the solution which is found to be proportional to the second-order time-averaged pressure gradient. If we time-average (1) for the solution given by (1), (2), (4), (5), (23), (24), (25), ((29a), (29b)), ((30a), (30b)), (39), and (41), we find that

(44) $$\begin{array}{c} c_{1}=R{\left(\;\frac{\bar{\partial p}}{\partial x}\;\right)}_{2}. \end{array}$$

The constant *c*_1_, which is related to the second-order pressure gradient distribution, may be obtained using ends conditions of the real physical problem.

The mean time average velocities may now be written as

(45) u−f=ε22Φf20′y=ε22Fy−F1+D−R∂p¯∂x21−y2+2Kn,

(46) u−p=ε22Φp20′y=ε22Gy−F1+D−R∂p¯∂x21−y2+2M+2Kn.

If no-slip, that is, Kn = 0, results of the present problems reduce exactly to the same as that found by L. M. Srivastava and V. P. Srivastava [5].

Also if no-slip, that is, Kn = 0 and the fluid is particles free, that is, *C* = 0, results of the present problems reduce exactly to the same as that found by Fung and Yih [34].

## 4. Numerical Results and Discussion

A close look at (45) reveals that the mean axial velocity of the fluid phase ${\overset{-}{u}}_{f}$ is dominated by the constant *D* and the parabolic distribution term −*R*(∂*p*/∂*x*)_2_(*l* − *y* ^2^ + 2Kn). The term *F*(*y*) − *F*(1), always a negative quantity, is negligible compared to *D*. The constant *D*, which initially arose from the slip condition of the axial velocity on the wall, is due to the value of Φ_*f*20_ at the boundary and is related to the mean velocity at the boundaries of the channel (at *y* = ±1) by $\overset{-}{u}=\left(\varepsilon^{2}/2\right)\left(D-{R\left(\partial p/\partial x\right)}_{2}\left(2Kn\right)\right)$. This shows that the slip boundary condition applies to the wavy wall and not to the mean position of the wall. It may be reminded here that the corresponding *D* does not appear in the particulate phase mean axial velocity as the particulate phase velocity at the walls was unspecified.

For the sake of comparison we define mean-velocity perturbation function J(y) in accordance with Fung and Yih [34] and L. M. Srivastava and V. P. Srivastava [5] as

(47) J=−200α2R2Fy−F1,

which gives mean time axial velocity of the fluid phase as

(48) u¯fy=ε22D−R∂p¯∂x21−y2+2Kn−α2R2200Jy.

It has been observed that urine, bacteria, or other materials some time pass from the* bladder* to the* kidney* or from one kidney to the other in direction opposite to the urine flow. Physiologists term these phenomena as “ureteral reflux.” Two different definitions of reflux exist in the literature; Shapiro et al. [40] call a flow reflux whenever there is a negative net displacement of a particle trajectory, while Yin and Fung [41] define a flow reflux whenever there is a negative mean velocity in the flow field. In the present analysis the latter definition of reflux is adopted as L. M. Srivastava and V. P. Srivastava [5].

Since *D* is always a positive quantity, $\overset{-}{u}=\left(\varepsilon^{2}/2\right)D$ at *y* = ±1 shows that the mean flow reversal will never occur at the boundaries. Further, from (48), it is clear that the reflux would occur when the mean pressure gradient (∂*p*/∂*x*)_2_ reaches a certain critical value. Thus, the critical reflux condition may be defined as one for which the mean velocity $\overset{-}{u}(y)$ is equal to* zero* on the center line *y* = 0; (48) yields

(49) C1R∂p¯∂x2  critical=1R1+2KnD−α2R2200J0.

For ${\left(\overset{-}{\partial p}/\partial x\right)}_{2}<{\left(\overset{-}{\partial p}/\partial x\right)}_{2\;\;critical\;\;reflux}$, there is no reflux and if ${\left(\overset{-}{\partial p}/\partial x\right)}_{2}>{\left(\overset{-}{\partial p}/\partial x\right)}_{2\;\;critical\;\;reflux}$ there will be reflux and a backward flow in the neighborhood of the center line occurs.

**Figure 2:**
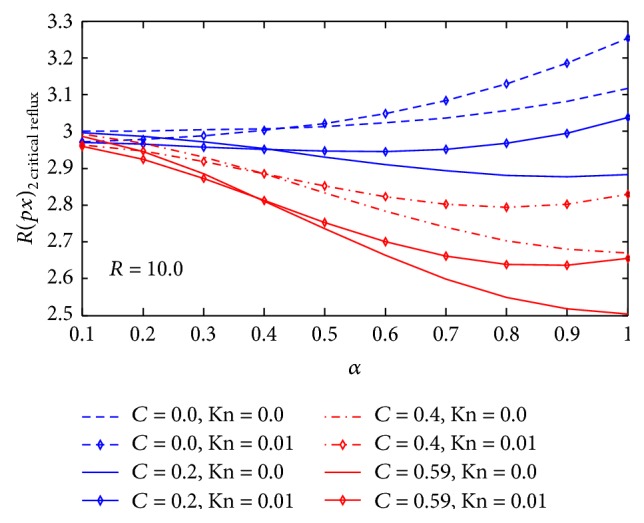
Effect of the Knudsen number Kn, particle concentration *C*, and wave number *α* on the critical reflux pressure gradient at *R* = 10.

The value of ${\left(\overset{-}{\partial p}/\partial x\right)}_{2\;\;critical\;\;reflux}$ for various values of *C*, *R*, Kn, and *α* is displayed in Figure 2. From the figure, the results reveal that the value of ${\left(\overset{-}{\partial p}/\partial x\right)}_{2\;\;critical\;\;reflux}$ with *y* for different values of *C*, for a fixed value of *R*, ${\left(\overset{-}{\partial p}/\partial x\right)}_{2\;\;critical\;\;reflux}$ decreases with increasing particle concentration *C*. However, *C* have significant influence over ${\left(\overset{-}{\partial p}/\partial x\right)}_{2\;\;critical\;\;reflux}$ only at higher values of *α*. In the presence of Knudsen number Kn, we observed that, at *α* ≤ 0.4, ${\left(\overset{-}{\partial p}/\partial x\right)}_{2\;\;critical\;\;reflux}$ decreases with increasing wave number *α*. However, *α* > 0.4 and ${\left(\overset{-}{\partial p}/\partial x\right)}_{2\;\;critical\;\;reflux}$ increases with increasing wave number *α*.

We observed that the critical reflux pressure ${\left(\overset{-}{\partial p}/\partial x\right)}_{2\;\;critical\;\;reflux}$ at a given *α* and *R* is lower for particle-fluid suspension than for particle-free fluid. This means that presence of particle in the fluid favors reversal flow.

**Figure 3:**
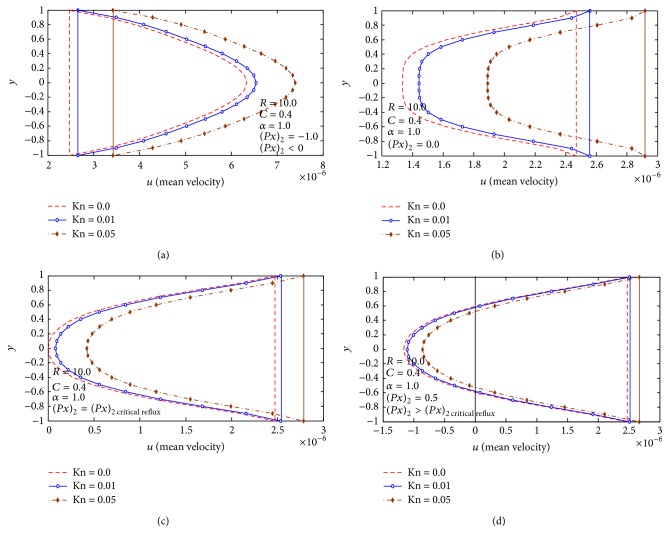
(a) Mean velocity distribution at (∂*p*/∂*x*)_2_ = −1.0. (b) Mean velocity distribution at (∂*p*/∂*x*)_2_ = 0.0. (c) Mean velocity distribution at (∂*p*/∂*x*)_2_ = (∂*p*/∂*x*)_2  Critical  reflux_. (d) Mean velocity distribution at (∂*p*/∂*x*)_2_ = 0.5.

**Figure 4:**
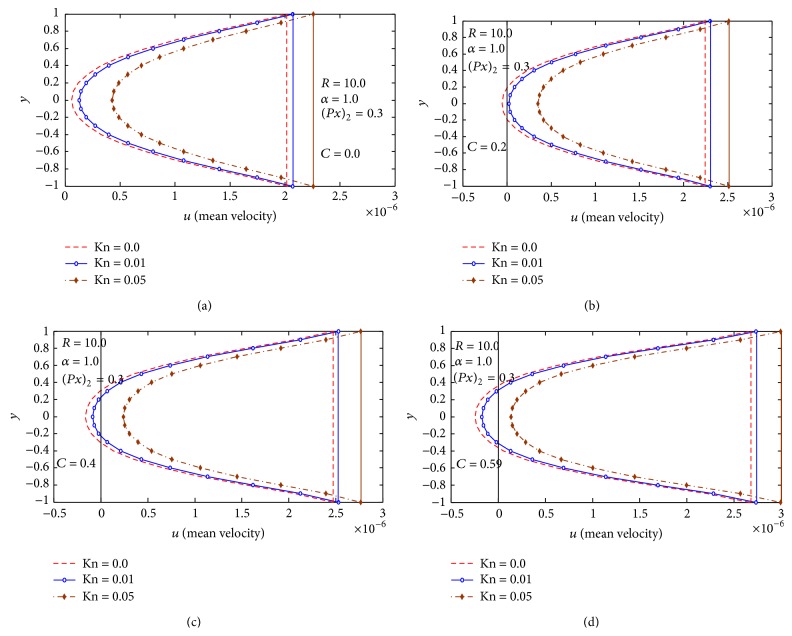
(a) Mean velocity distribution at *C* = 0.0, *α* = 1.0, ${\left(\overset{-}{\partial p}/\partial x\right)}_{2}=0.3$, and *R* = 10. (b) Mean velocity distribution at *C* = 0.2, *α* = 1.0, ${\left(\overset{-}{\partial p}/\partial x\right)}_{2}=0.3$, and *R* = 10. (c) Mean velocity distribution at *C* = 0.4, *α* = 1.0, ${\left(\overset{-}{\partial p}/\partial x\right)}_{2}=0.3$, and *R* = 10. (d) Mean velocity distribution at *C* = 0.59, *α* = 1.0, ${\left(\overset{-}{\partial p}/\partial x\right)}_{2}=0.3$, and *R* = 10.

**Figure 5:**
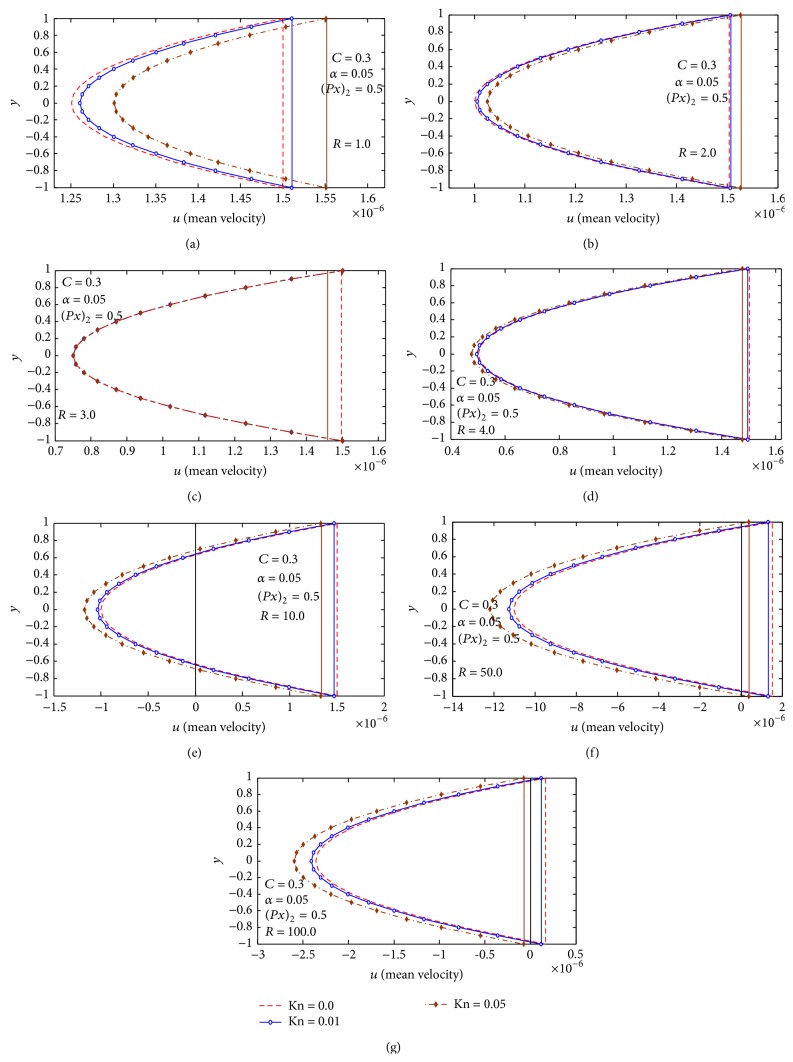
(a) Mean velocity distribution at *R* = 1.0, *α* = 0.05, ${\left(\overset{-}{\partial p}/\partial x\right)}_{2}=0.5$, and *C* = 0.3. (b) Mean velocity distribution at *R* = 2.0, *α* = 0.05, ${\left(\overset{-}{\partial p}/\partial x\right)}_{2}=0.5$, and *C* = 0.3. (c) Mean velocity distribution at *R* = 3.0, *α* = 0.05, ${\left(\overset{-}{\partial p}/\partial x\right)}_{2}=0.5$, and *C* = 0.3. (d) Mean velocity distribution at *R* = 4.0, *α* = 0.05, ${\left(\overset{-}{\partial p}/\partial x\right)}_{2}=0.5$, and *C* = 0.3. (e) Mean velocity distribution at *R* = 10.0, *α* = 0.05, ${\left(\overset{-}{\partial p}/\partial x\right)}_{2}=0.5$, and *C* = 0.3. (f) Mean velocity distribution at *R* = 50.0, *α* = 0.05, ${\left(\overset{-}{\partial p}/\partial x\right)}_{2}=0.5$, and *C* = 0.3. (g) Mean velocity distribution at *R* = 100.0, *α* = 0.05, ${\left(\overset{-}{\partial p}/\partial x\right)}_{2}=0.5$, and *C* = 0.3.

**Figure 6:**
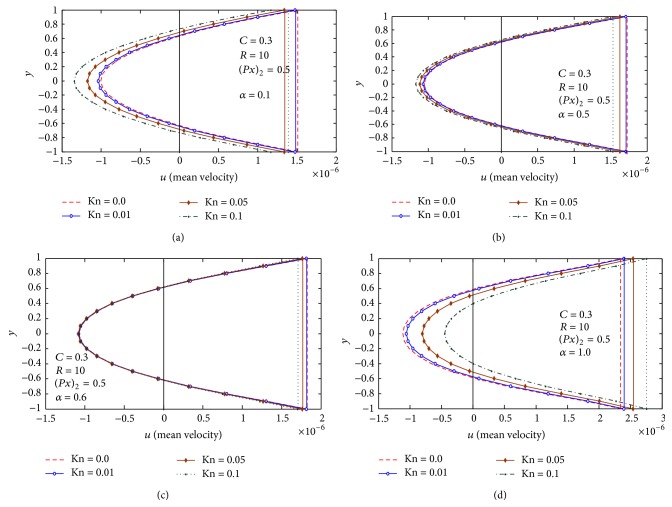
(a) Mean velocity distribution at *α* = 0.1,$\;{\left(\overset{-}{\partial p}/\partial x\right)}_{2}=0.5$, and *C* = 0.3. (b) Mean velocity distribution at *α* = 0.5,$\;{\left(\overset{-}{\partial p}/\partial x\right)}_{2}=0.5$, and *C* = 0.3. (c) Mean velocity distribution at *α* = 0.6,$\;{\left(\overset{-}{\partial p}/\partial x\right)}_{2}=0.5$, and *C* = 0.3. (d) Mean velocity distribution at *α* = 1.0,$\;{\left(\overset{-}{\partial p}/\partial x\right)}_{2}=0.5$, and *C* = 0.3.

Finally, in Figures 3–6, the mean-velocity distribution with reversal flow is displayed. Effects of *C*, *R*, Kn, and ${\left(\overset{-}{\partial p}/\partial x\right)}_{2}$ on mean velocity and reversal flow are shown.

**Figure 7:**
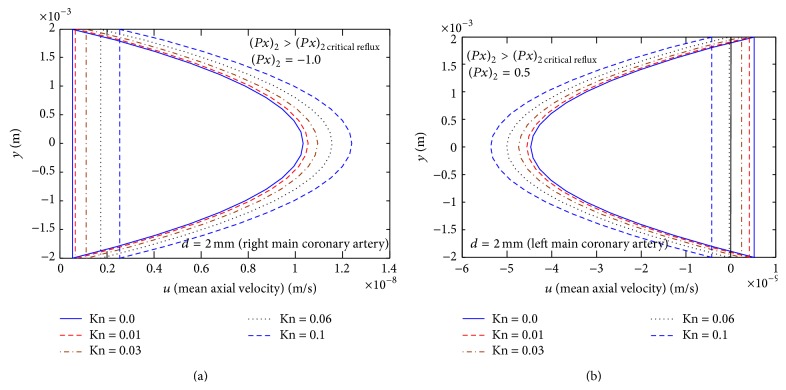
(a) Mean axial velocity (m/s) distribution at ${\left(\overset{-}{\partial p}/\partial x\right)}_{2}<{\left(\overset{-}{\partial p}/\partial x\right)}_{2\;critical\;reflux}$(${\left(\overset{-}{\partial p}/\partial x\right)}_{2}=-1.0$). (b) Mean axial velocity (m/s) distribution at$\;{\left(\overset{-}{\partial p}/\partial x\right)}_{2}>{\left(\overset{-}{\partial p}/\partial x\right)}_{2\;critical\;reflux}$ ${\left((\overset{-}{\partial p}/\partial x\right)}_{2}=0.5)$.

Figure 7 studies the effect of Knudsen number Kn and the mean second-order pressure gradient (∂*p*/∂*x*)_2_ on the mean-velocity distribution and reversal flow for *C* = 0.4, *α* = 1.0, and *R* = 10; we notice that the mean-velocity distribution increases with increasing Knudsen number Kn forward for ${\left(\overset{-}{\partial p}/\partial x\right)}_{2}<{\left(\overset{-}{\partial p}/\partial x\right)}_{2\;\;critical\;\;reflux}$ while for ${\left(\overset{-}{\partial p}/\partial x\right)}_{2}>{\left(\overset{-}{\partial p}/\partial x\right)}_{2\;\;critical\;\;reflux}$ the velocity increases the reflux flow. Also we notice that the mean-velocity distribution decrease with increasing the value of ${\left(\overset{-}{\partial p}/\partial x\right)}_{2}$.

Also we notice that the value of ${\left(\overset{-}{\partial p}/\partial x\right)}_{2\;\;critical\;\;reflux}$ decreases by increasing the Knudsen number Kn.

Figure 4 studies the effect of Knudsen number Kn and particle concentration *C* on the mean-velocity distribution mean and reversal flow for *α* = 1.0, ${\left(\overset{-}{\partial p}/\partial x\right)}_{2}=0.3$, and *R* = 10, and the figures reveal that the reversal flow increases with increasing particle concentration *C*, but the presence of Knudsen number Kn results in a decrease in the reversal flow. Also we notice that the mean-velocity distribution increases with increasing Kn. Interpreted physiologically, this means that, under the same conditions, urine in which solute particles are suspended (i.e., urine from a diseased kidney) is more susceptible to reversal flow in ureter, in comparison to pure urine without solute particles.

Figure 5 studies the effect of Knudsen number Kn and the Reynolds number *R* on the mean-velocity distribution and reversal flow for *α* = 0.05, ${\left(\overset{-}{\partial p}/\partial x\right)}_{2}=0.5$, and *C* = 0.3, and the figures reveal that the reversal flow increases with increasing Reynolds number *R*. Also we notice that from Figures 5(a) and 5(b), at *R* < 3, the presence of Knudsen number Kn results a decrease in the mean-velocity distribution, from Figure 5(c) at *R* = 3, the effect of Knudsen number vanishes, with increasing Kn at *R* > 3, and we observed that the presence of Knudsen number Kn results in increase in the mean-velocity distribution and the reversal flow.  Figure 6 studies the effect of Knudsen number Kn and the wave number *α* on the mean-velocity distribution and reversal flow for *R* = 10, ${\left(\overset{-}{\partial p}/\partial x\right)}_{2}=0.5$, and *C* = 0.3; the figures reveal that the reversal flow increases with increasing wave number *α*. Also we notice that from Figures 6(a) and 6(b), at *α* < 0.6, the presence of Knudsen number Kn results in an increase in the reversal flow, from Figure 6(c) at *α* = 0.6, the effect of Knudsen number vanishes, with increasing *α* at *α* > 0.6, and we observed that the presence of Knudsen number Kn results in decrease in the reversal flow.

Next, we return to the dimensional flow problem; the dimensional mean axial velocity 〈*u*_*f*_〉 is equal to the dimensionless mean net axial velocity ${\overset{-}{u}}_{f}$ as given by (48) multiplied by the factor *c*. The properties of the blood are given by *ρ*_*f*_ = 1066 kg/m3 and *μ* = 4 × 10^−3^ Nm^−2^ s. The particle concentration *C* various accurate up to *C* = 0.6 [39]. The frequency *f* of the wave is related to the wave speed *c* and the wavelength *λ* according to *λ* = *c*/*f*.

According to the Knudsen number, the flow regimes can be divided into various regions. These are continuum, slip, transition, and free molecular flow regimes. If Kn < 0.001, so that molecular mean free path of the molecules is negligible in comparison to the geometrical dimensions, the fluid can be treated as a continuous medium. If 0.001 < Kn < 0.1, it is found that the fluid loses grip on the boundaries and tends to slip along the walls of the domain. If 0.1 < Kn < 3.0, it is transition flow regimes. Finally, the flow enters the free molecular regime when Kn > 3.0, each requiring a particular type of analysis [21, 23].

For example, for the left main coronary artery, the range of the diameter is 2.0–5.5 mm (mean 4 mm) and wavelength range *λ* = 2.512–12.56 cm [39]. The dimensional mean axial velocity 〈*u*_*f*_〉 (m/s) is plotted versus *y*(*m*), for *λ* = 12.56 cm, the half of mean width *d* = 2.0 mm, the wave has amplitude *a*_0_ = 10^−4^ mm, and *C* = 0.3, with various values of Knudsen number Kn, for Kn = 0.0, Kn = 0.01, Kn = 0.03, Kn = 0.06, and Kn = 0.1. We observe that, from Figure 7(a), for ${\left(\overset{-}{\partial p}/\partial x\right)}_{2}<{\left(\overset{-}{\partial p}/\partial x\right)}_{2\;critical\;reflux}$(${\left(\overset{-}{\partial p}/\partial x\right)}_{2}=-1.0$), the mean axial velocity 〈*u*_*f*_〉 increases with increasing Knudsen number Kn and there is no reflux flow forward for ${\left(\overset{-}{\partial p}/\partial x\right)}_{2}<{\left(\overset{-}{\partial p}/\partial x\right)}_{2\;critical\;reflux}$ while, from Figure 7(b), for ${\left(\overset{-}{\partial p}/\partial x\right)}_{2}>{\left(\overset{-}{\partial p}/\partial x\right)}_{2\;critical\;reflux}$ ${\left((\overset{-}{\partial p}/\partial x\right)}_{2}=0.5)$ there will be reflux flow and a backward flow in the neighborhood of the center line occurring, and the mean axial velocity 〈*u*_*f*_〉 increases in the reversal flow with increasing Knudsen number Kn.

## 5. Conclusions 

There is not any attempt to study the effect of slip conditions on the flow of a particle-fluid suspension with peristalsis. The purpose of this paper is to study the slip effects on the peristaltic pumping of a particle-fluid mixture in a two-dimensional channel. It is an application of the two-dimensional analysis of peristaltic motion of a particle-fluid mixture by L. M. Srivastava and V. P. Srivastava [5] and the two-dimensional analysis of peristaltic motion of single phase fluid by Fung and Yih [34] in the presence of slip effects. The mathematical model considers a particle-fluid mixture between infinite parallel walls with slip condition on which a sinusoidal traveling wave is imposed. A perturbation solution is obtained which satisfies the momentum equations for the case in which amplitude ratio (wave amplitude/channel half width) is small. Finally, the phenomenon of the mean flow reversal is presented and its physiological implication is discussed. Beside the engineering applications of pumping particle-fluid mixture by peristalsis, the present analysis of two-phase flow process is potentially important in regard to biofluid transport by peristalsis muscular contractions in body organs where fluids behave like particle-fluid mixtures, namely, chime in small intestine, spermatic fluid in cervical canal, urine (from a diseased kidney) in ureter, and blood suspension in arteriole.

Some concluding remarks are as follows.

(i) The reversal flow increases with increasing particle concentration *C*, but the presence of Knudsen number Kn results in a decrease in the reversal flow. The presence of Knudsen number Kn results in a decrease in the reversal flow.
(ii) Also we notice that the mean-velocity distribution increases with increasing Kn. Interpreted physiologically, this means that, under some conditions, urine in which solute particles are suspended (i.e., urine from a diseased kidney) is more susceptible to reversal flow in ureter, in comparison to pure urine without solute particles.
(iii) For example, for the left main coronary artery, the mean axial velocity 〈*u*_*f*_〉 increases with increasing Knudsen number Kn and there is no reflux flow forward for ${\left(\overset{-}{\partial p}/\partial x\right)}_{2}<{\left(\overset{-}{\partial p}/\partial x\right)}_{2\;critical\;reflux}$ while for ${\left(\overset{-}{\partial p}/\partial x\right)}_{2}>{\left(\overset{-}{\partial p}/\partial x\right)}_{2\;critical\;reflux}$ there will be reflux and a backward flow in the neighborhood of the center line occurring, and the mean axial velocity 〈*u*_*f*_〉  increases in the reversal flow with increasing Knudsen number Kn.

Comparing with other models for verifications of results, the present model gives the most general form of velocity expression from which the other mathematical models can easily be obtained by proper substitutions. It is of interest to note that the result of the present model includes results of different mathematical models such as the following.

(1) The results of L. M. Srivastava and V. P. Srivastava [5] have been recovered by taking Knudsen number kn = 0.0 (no-slip condition).
(2) The results of Fung and Yih [34] have been recovered by taking Knudsen number kn = 0.0 and the fluid is particles-free; that is, *C* = 0.
